# Retinal hypoplasia and degeneration result in vision loss in Friedreich ataxia

**DOI:** 10.1002/acn3.51830

**Published:** 2023-06-19

**Authors:** Layne N. Rodden, Kellie McIntyre, Medina Keita, Mckenzie Wells, Courtney Park, Victoria Profeta, Amy Waldman, Christian Rummey, Laura J. Balcer, David R. Lynch

**Affiliations:** ^1^ Departments of Pediatrics and Neurology, Children's Hospital of Philadelphia, Perelman School of Medicine University of Pennsylvania Philadelphia Pennsylvania USA; ^2^ Clinical Data Science GmbH Basel Switzerland; ^3^ Departments of Neurology, Population Health and Ophthalmology NYU Grossman School of Medicine New York New York USA

## Abstract

**Objective:**

Friedreich ataxia (FRDA) is an inherited condition caused by a GAA triplet repeat (GAA‐TR) expansion in the *FXN* gene. Clinical features of FRDA include ataxia, cardiomyopathy, and in some, vision loss. In this study, we characterize features of vision loss in a large cohort of adults and children with FRDA.

**Methods:**

Using optical coherence tomography (OCT), we measured peripapillary retinal nerve fiber layer (RNFL) thickness in 198 people with FRDA, and 77 controls. Sloan letter charts were used to determine visual acuity. RNFL thickness and visual acuity were compared to measures of disease severity obtained from the Friedreich Ataxia Clinical Outcomes Measures Study (FACOMS).

**Results:**

The majority of patients, including children, had pathologically thin RNFLs (mean = 73 ± 13 μm in FRDA; 98 ± 9 μm in controls) and low‐contrast vision deficits early in the disease course. Variability in RNFL thickness in FRDA (range: 36 to 107 μm) was best predicted by disease burden (GAA‐TR length X disease duration). Significant deficits in high‐contrast visual acuity were apparent in patients with an RNFL thickness of ≤68 μm. RNFL thickness decreased at a rate of −1.2 ± 1.4 μm/year and reached 68 μm at a disease burden of approximately 12,000 GAA years, equivalent to disease duration of 17 years for participants with 700 GAAs.

**Interpretation:**

These data suggest that both hypoplasia and subsequent degeneration of the RNFL may be responsible for the optic nerve dysfunction in FRDA and support the development of a vision‐directed treatment for selected patients early in the disease to prevent RNFL loss from reaching the critical threshold.

## Objective

Friedreich ataxia (FRDA) is most commonly (>96%) caused by a GAA triplet repeat (GAA‐TR) expansion in intron 1 of the *FXN* gene.^1^, ^2^ Most people have less than nine GAA triplets on both *FXN* genes, but an expansion of 40 to 1500 triplets on both alleles results in FRDA. Less than 4% of people with FRDA carry an expansion on one allele and a point mutation or a deletion on the other. Heterozygous carriers of one pathogenic allele are asymptomatic throughout life. Clinical features include ataxia, cardiomyopathy, scoliosis, and diabetes.^3^, ^4^, ^5^ Vision loss and retinal pathology can occur later in FRDA and in idiosyncratic individuals earlier in disease, but the quantification and temporal course of such features remain understudied.^6^, ^7^, ^8^, ^9^, ^10^, ^11^, ^12^, ^13^, ^14^, ^15^ Furthermore, at present no methodology exists for predicting eventual visual loss in those at risk.

FRDA is a progressive disease, and clinical features worsen over time.^3^, ^4^, ^5^ The length of the GAA‐TR especially the ones in the shorter of the two alleles (denoted “GAA1”) predicts age of onset and symptom severity; those with longer GAA‐TR have earlier onset and more severe disease.^2^, ^16^, ^17^, ^18^ There is generally no difference in symptomatology between males vs. females, but some studies have found modest differences in some measures of speed of disease progression showing a faster progression of some neurological symptoms in females.^18^ The modified Friedreich Ataxia Rating Scale (mFARS) and FA Functional Disease Stage score provide two validated metrics of neurological severity and can detect temporal progression of the disease. mFARS correlates with both GAA1 length and disease duration but is best predicted by the product of the two, called disease burden.^16^, ^17^, ^18^, ^19^, ^20^, ^21^, ^22^ This measure predicts many features of FRDA better than its two components alone.^22^ Similarly, in linear regression models, outcome measures that capture progression are predicted by both disease duration and GAA repeat length, such that those with longer GAA1 lengths and longer disease duration have more severe disease including visual acuity deficits in some cases. In such individuals, central vision is usually spared until later in disease, while loss of low‐contrast vision occurs much earlier.^6^

*Visual quality of life measures suggest that both afferent and high‐contrast visual loss are directly noted within the complex symptomatology of FA. Clinical symptoms of vision loss may go unnoticed early in the disease by the patient except when asked to perform low‐contrast visual acuity letter chart tests*.^6^, ^12^, ^23^ *Once significant vision loss starts to become noticeable by patients, they usually have developed at least some deficit on high‐contrast vision charts*. In FRDA, visual decline largely reflects optic neuropathy with loss of the RNFL rather than macular involvement; retinal pigmented epithelial cells are not affected.^6^ While we and others have found that visual function from optic neuropathy worsens over time in FRDA, the quantitative links of functional vision to loss of the RNFL characteristic of optic neuropathy are not well defined.^6^, ^7^, ^8^, ^9^, ^10^, ^11^, ^12^, ^13^, ^14^, ^15^ In this study, we used optical coherence tomography (OCT) to investigate the changes in the RNFL in a diverse cohort of participants with FRDA, including young children, and whether such changes contribute to vision loss.

## Methods

### Participants and samples

All protocols for collection of data were approved by the Institutional Review Board at the Children's Hospital of Philadelphia. Informed consent was obtained before participation. Genetic confirmation with GAA repeat length determination was obtained on all subjects via commercial or research testing. We collected cross‐sectional data from an ongoing natural history study called Friedreich Ataxia Clinical Outcome Measure Study (FACOMS).^16^, ^17^, ^18^, ^19^, ^20^ FACOMS contains data on GAA‐TR length, age at data collection, age of onset, mFARS scores, FA Functional Disease Stage scores, high‐contrast and low‐contrast letter acuity testing via Sloan letter charts. Disease burden was calculated as length of the shorter GAA allele (GAA1) multiplied by disease duration attachment.^22^

### Optical coherence tomography

OCT scans were performed on a Cirrus OCT machine (Carl Zeiss Meditec, Dublin, CA) with the Optic Disc Cube 200 × 200 program and Optic Nerve Head (ONH) and Retinal Nerve Fiber Layer (RNFL) Oculus Uterque (OU) Analysis via on‐device Cirrus software.^6^ Scans of both eyes were attempted on participants to evaluate RNFL thickness. Data were included from participants with at least one usable eye scan per timepoint: *n* = 198 people with FRDA including *n* = 32 participants with follow‐up scans at multiple time points. Each scan taken from the same person at different time points (at least 1 year apart) was treated as a separate data point. Sixty‐three of the present participants were reported previously and 40 others in a parallel study.^6^ Scans from 11 participants (all with FRDA) were excluded due to low quality, and scans from 1 participant with FRDA were excluded due to a confounding retinal disease unrelated to FRDA. To establish that the findings were independent of the technical aspects, we assessed a second smaller group, a confirmational cohort (*n* = 45: 16 non‐FRDA controls, 14 carriers, 15 FRDA patients), using a Spectralis OCT machine (Heidelberg) at a different site.

Controls were provided in multiple manners. Cirrus OCT provides an internal control dataset ranking RNFL values by age‐adjusted percentiles in participants age ≥ 18. In addition, we utilized a separate set of pediatric controls obtained from a parallel study on multiple sclerosis in children. Finally, we performed OCT on a limited number of controls and carriers in parallel with participants with FRDA.

### Statistical analyses

Data analysis was performed using STATA SE/17 software and Prism9. Linear regressions and Student's *t*‐tests were used where appropriate.

## Results

### RNFLs are thin in children and adults with FRDA

**Figure 1:**
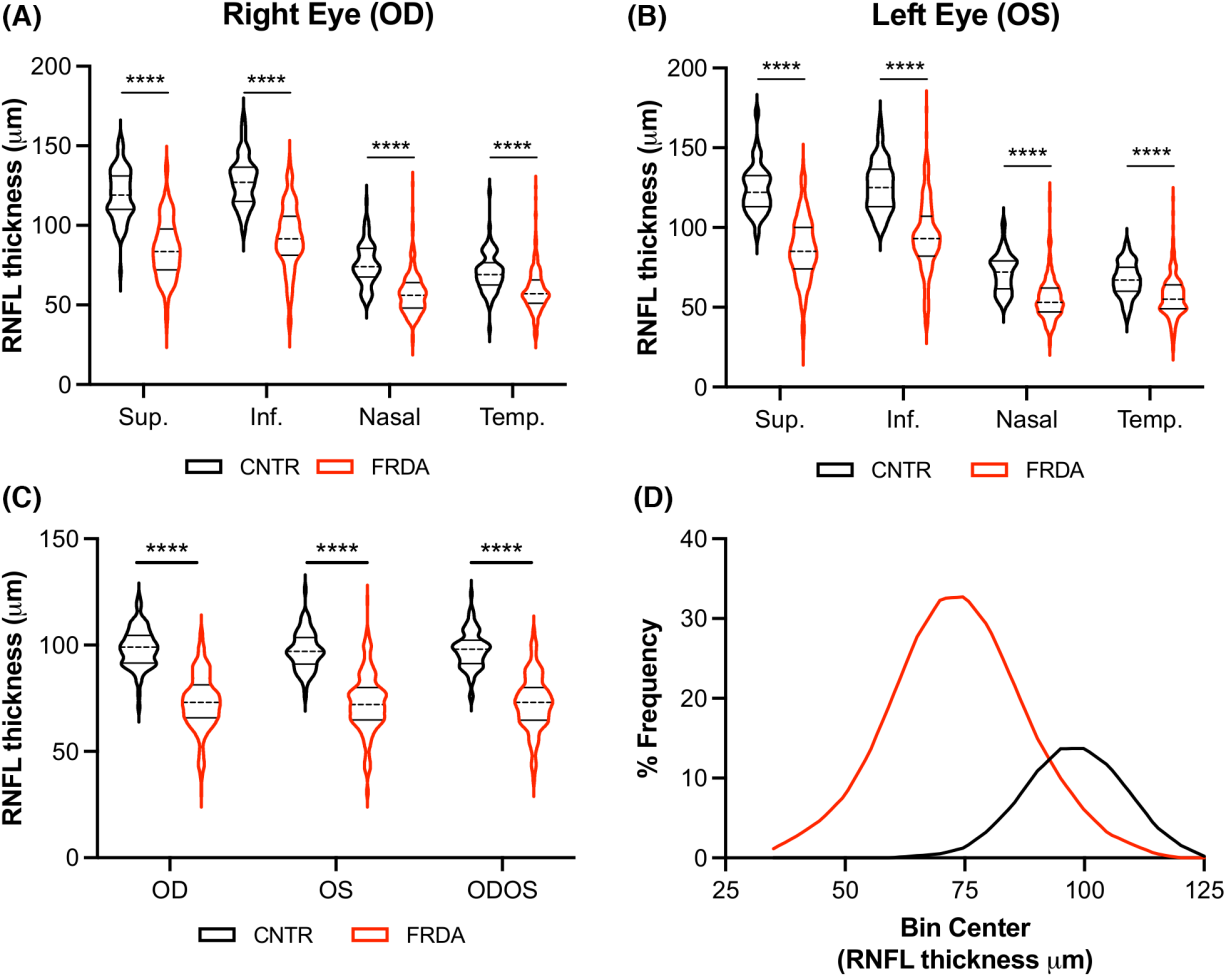
RNFL thickness is reduced in FRDA. RNFL thickness in the 4 quadrants of (A) the right eye (OD) and (B) the left eye (OS) in 77 controls and 198 participants with FRDA. (C) Average RNFL thickness of all quadrants in the right eye (OD), left eye (OS), and the average of OD and OD (ODOS) in 77 controls and 198 participants with FRDA. (D) Frequency distributions of RNFL thickness controls (black curve) and participants with FRDA (red curve). Sup. = superior, Inf. = inferior, Temp. = temporal. Stats: *t*‐test, ****p < 0.0001.

**Figure 2:**
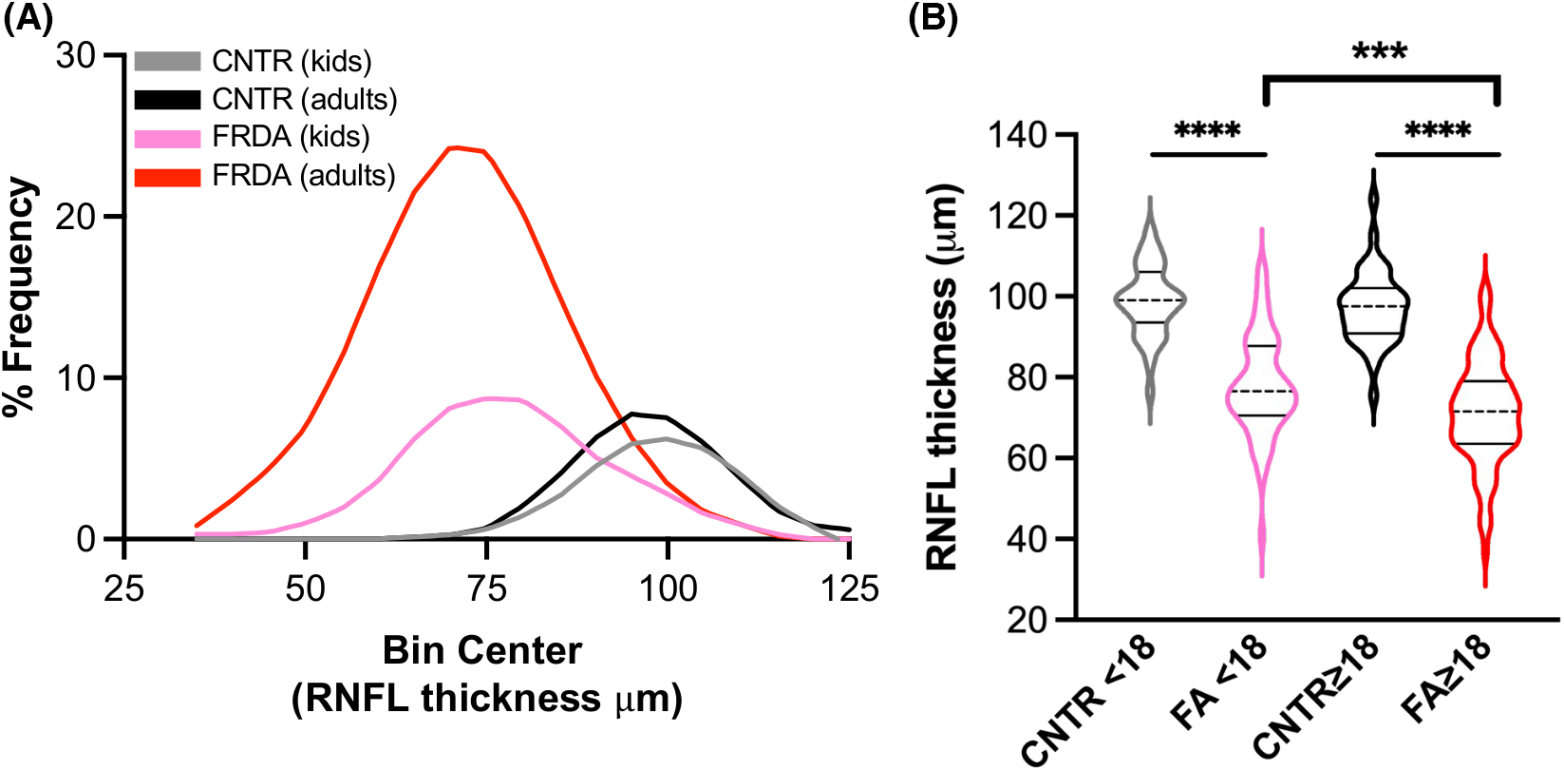
Children with FRDA have thin RNFLs early in disease. (A) Frequency distributions of RNFL thickness in kids (<18 years *n* = 35 CNTR, *n* = 61 FRDA) and adults (≥18 years *n* = 42 CNTR, *n* = 172 FRDA). Black and gray curves = non‐FRDA controls, pink and red curves = FRDA. (B) RNFL thickness plotted by age and FRDA status. Stats: one‐way ANOVA with Tukey's post hoc, ****p < 0.0001, ***p < 0.001.

Using Cirrus OCT technology, retinas of 198 people with FRDA, 8 heterozygous carriers, and 69 controls were scanned to visualize the optic nerve and RNFL thickness. The OCT procedure showed a standard deviation of 0.9 μm across three consecutive scans (taken less than 1 year apart) for four control participants and 1.6 μm across 2 consecutive scans in 15 participants with FRDA (Fig. S1), demonstrating reproducibility. The participants with FRDA in this study displayed representative demographics of the FRDA population with a wide range of GAA‐TR lengths, ages, and ages of onset (Table 1). Heterozygous carriers of one GAA‐TR showed no significant difference in RNFL thickness compared to non‐FRDA controls (Fig. S2); therefore, hereafter controls in this study consist of both non‐FRDA and heterozygous carriers and are referred to collectively as controls. RNFLs were significantly thinner in people with FRDA compared to controls in all four quadrants of the retina (superior, inferior, nasal, and temporal) in both the right eye (OD) and left eye (OS) (Fig. 1A–C). We found no significant difference in any quadrant between the right eye and left in either controls or participants with FRDA, suggesting a symmetrical pathology (Table S1). Control participants had an average RNFL thickness of 98 μm with a range of 76–124 μm, and participants with FRDA had an average RNFL thickness of 73 μm with a range of 36 to 107 μm (Fig. 1D). Thirty‐one percent (*n* = 61) of the cohort was <18 years of age, and when analyzed separately from adults with FRDA, children also showed significantly thin RNFLs (Fig. 2A,B).

**Table 1:** Participant demographics.

*n* = 198 participants with FRDA	
Female (%)	56%
Age^1^ (median ± SD, range) (*n* = 232)	24 ± 13 (8–78)
GAA1^2^ (median ± SD, range) (*n* = 221)	673 ± 238 (41–1275)
GAA2^2^ (median ± SD, range) (*n* = 221)	1000 ± 228 (170–1600)
AoO^1^ (median ± SD, range) (*n* = 228)	11 ± 9 (2–63)

All values were calculated using available data from the *n* = 198 participants with FRDA which included (for *n* = 32 participants) multiple scans taken at different time points.

^1^ Years.

^2^ Number of triplets.

**Figure 3:**
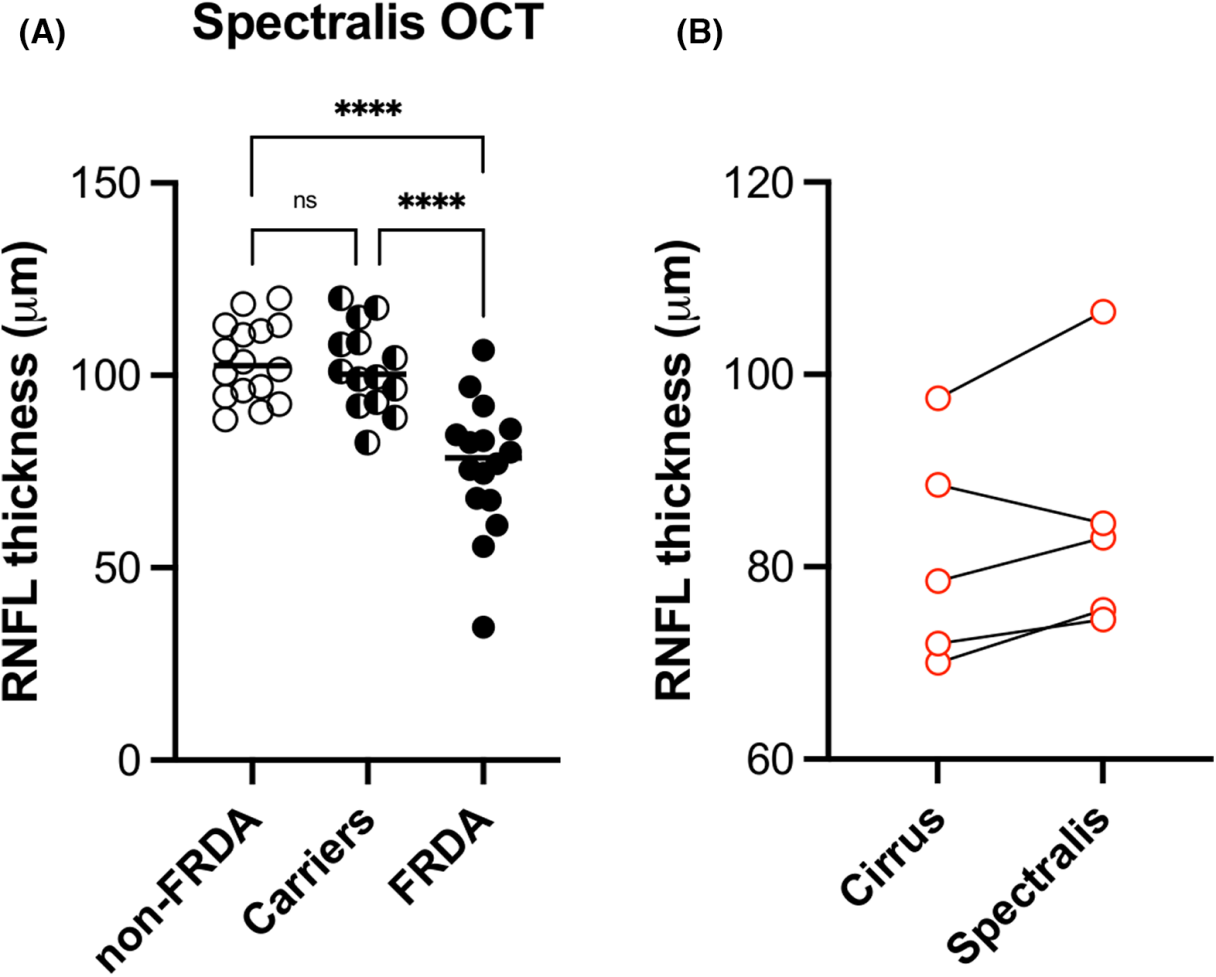
RNFL thickness measured with two different OCT devices give similar outcomes. (A) RNFL thickness for non‐FRDA controls, carriers, and participants with FRDA. (B) comparison of RNFL thickness values for five participants that were assessed by both machines. Stats: one‐way ANOVA with Tukey's post hoc analysis. n.s. = not significant, ****p < 0.0001.

To establish that the findings were independent of the technical aspects, we assessed a second smaller group, a confirmational cohort (*n* = 45: 16 non‐FRDA controls, 14 carriers, 15 FRDA patients), using a Spectralis OCT machine (Heidelberg) at a different site. Again, controls and carriers had identical average values of 104 μm with FRDA patients having a value of 77 μm (Fig. 3A). Interestingly, of the five participants (with FRDA) studied in both groups (Cirrus and Spectralis), all but one had slightly higher values by Spectralis OCT (Fig. 3B). In addition, the Spectralis OCT machine captured data on one subject unable to be assessed technically by the Cirrus OCT machine (average RNFL for both eyes = 34.5 μm). Overall, the results were similar across both the Cirrus and Spectralis cohorts.

### RNFL thickness is predicted by disease burden

**Figure 4:**
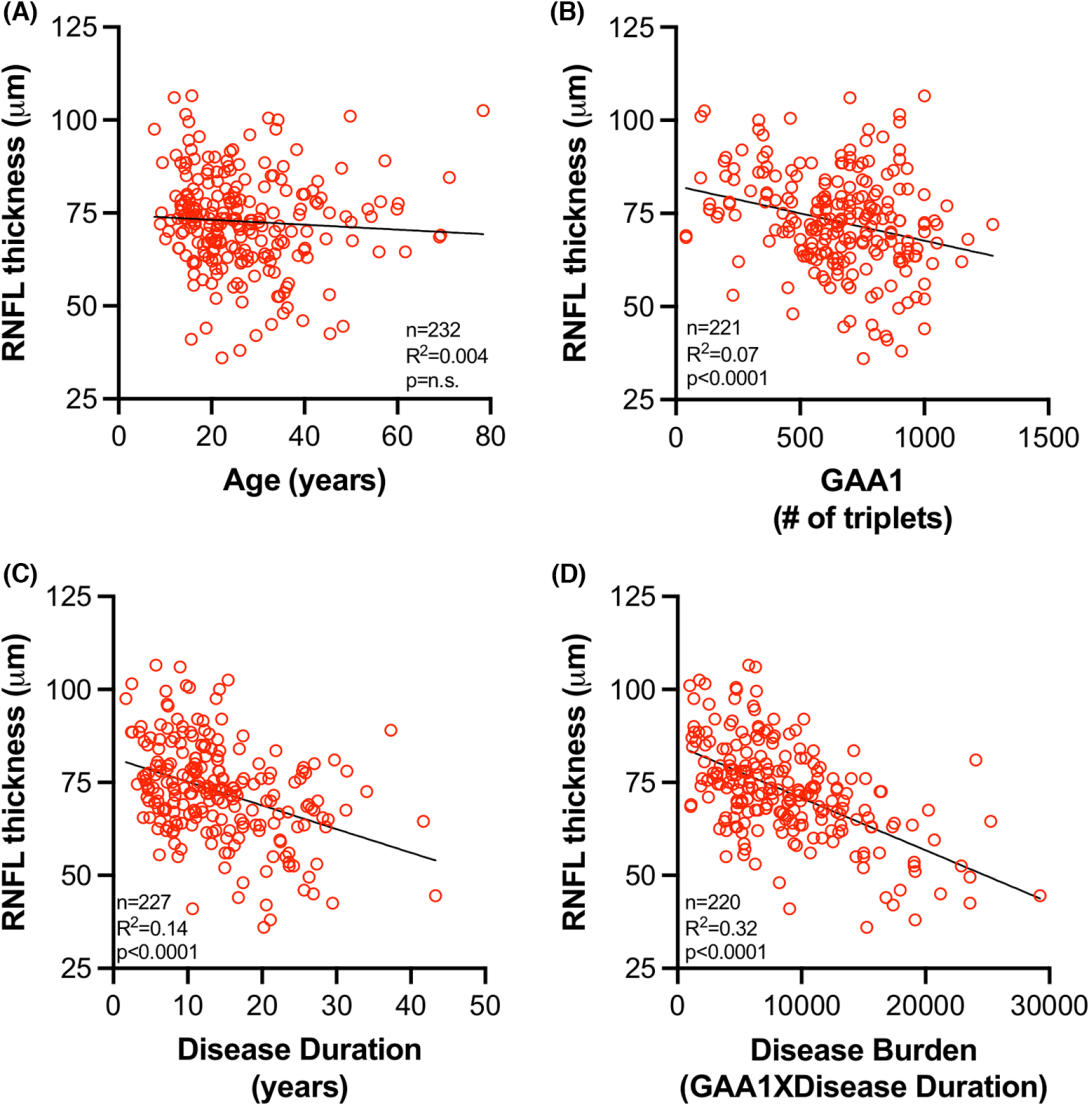
Disease burden predicts RNFL thickness. RNFL thickness plotted against (A) age, (B) GAA1 length, (C) disease duration [age minus age of onset], and (D) disease burden [GAA1 multiplied by disease duration].

To determine whether RNFL thickness correlated with predictors of FRDA disease progression, average RNFL thickness across both eyes was compared to several parameters independently: age, disease duration, length of the shorter GAA‐TR (GAA1), or disease burden (the product of disease duration and GAA1). There was no significant correlation in a linear regression model between RNFL thickness and age (Fig. 4A). There was a modest correlation between RNFL thickness and either GAA1 length (*R* ^2^ = 0.12, *p* < 0.0001) or disease duration (*R* ^2^ = 0.14, *p* < 0.0001), but disease burden was the best predictor of RNFL thickness (*R* ^2^ = 0.32, *p* < 0.0001) (Fig. 4B–D). Like many other measures of neurological function in FRDA, the degree of pathophysiology in the retina is a consequence of both time and GAA1 length. An analysis of RNFL thickness based on sex showed that males had modestly but significantly thinner RNFLs (mean 70 μm for males vs 75 μm for women, *t*‐test, *p* = 0.01), indicating that sex may also contribute to variability in RNFL thickness.

To confirm this association with the disease burden metric, we used linear multiple regression analysis to investigate the ability of GAA1 length, disease duration, and sex to predict RNFL thickness. Multiple regression analysis gave similar results: RNFL thickness Vs multiple variables collectively (GAA1, disease duration, and sex): *p* < 0.0001, *R* ^2^ = 0.34 (individual *p* values <0.001 for GAA1, <0.001 for disease duration, and *p* = 0.001 for sex). While men had thinner RNFLs, no differences in disease duration or GAA1 length were found between men and women, further suggesting that sex is another variable that contributes to RNFL thickness.

### RNFL thickness predicts severity of neurological and visual deficits in FRDA

**Figure 5:**
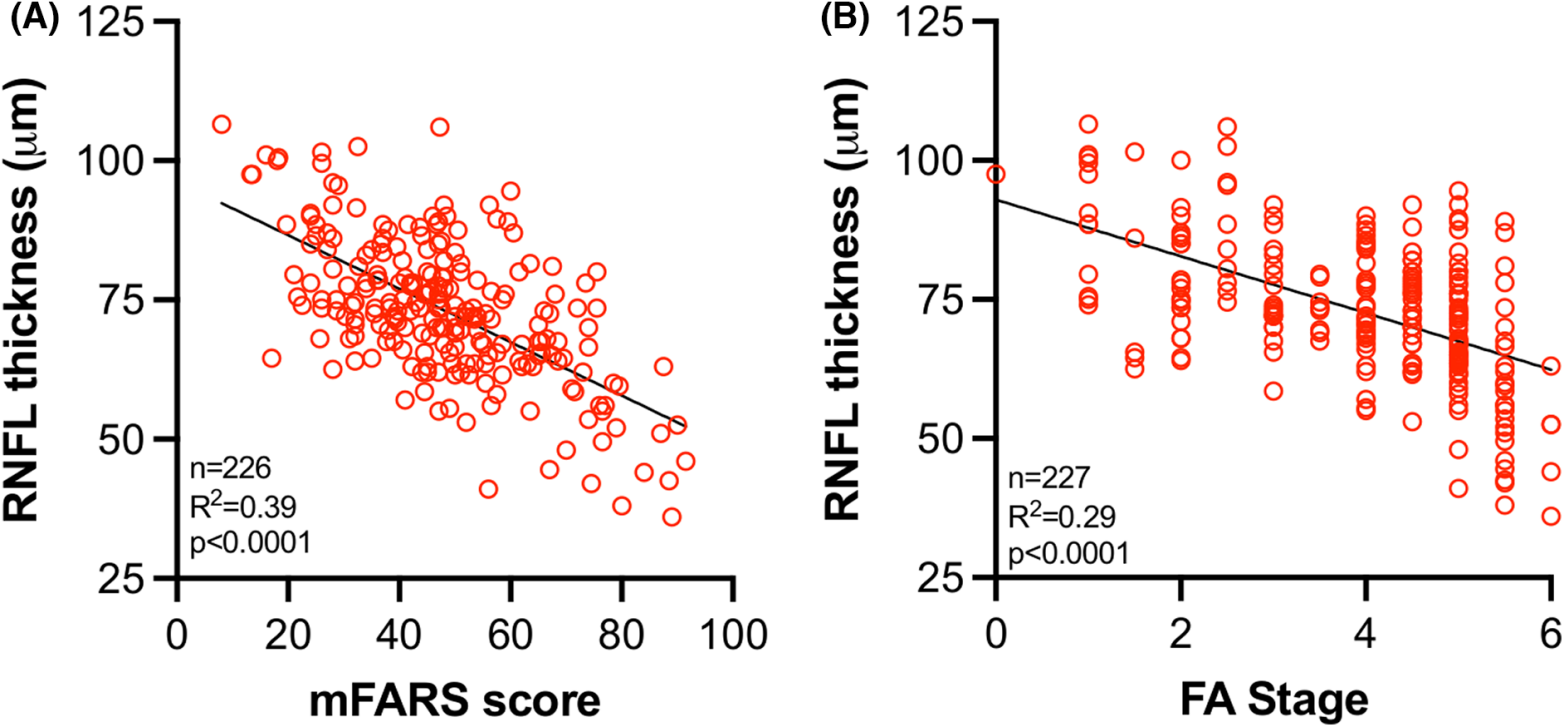
RNFL thickness correlates with neurological severity in FRDA. RNFL thickness plotted against (A) mFARS score and (B) FA Functional Disease Stage (FA stage).

RNFL thickness significantly predicted both mFARS score (*R* ^2^ = 0.43, *p* < 0.0001) and FA Functional Disease Stage (*R* ^2^ = 0.29, *p* < 0.0001) (Fig. 5A,B), two validated and commonly used measures of neurological progression in FRDA. The mFARS examination can be subdivided into upright stability, bulbar, upper limb coordination, and lower limb coordination components and some studies have shown that different subcomponents of mFARS and other neurological examinations may be more reliable than others or may better predict certain disease features.^16^, ^24^ To see whether this was the case in our study, we compared RNFL thickness to the upright stability subscore of the mFARS with linear regression (*n* = 158, *R* ^2^ = 0.29, *p* < 0.0001) (Fig. S3). The upright stability subcomponent significantly predicted RNFL thickness albeit with a weaker correlation coefficient than did the composite mFARS score.

**Figure 6:**
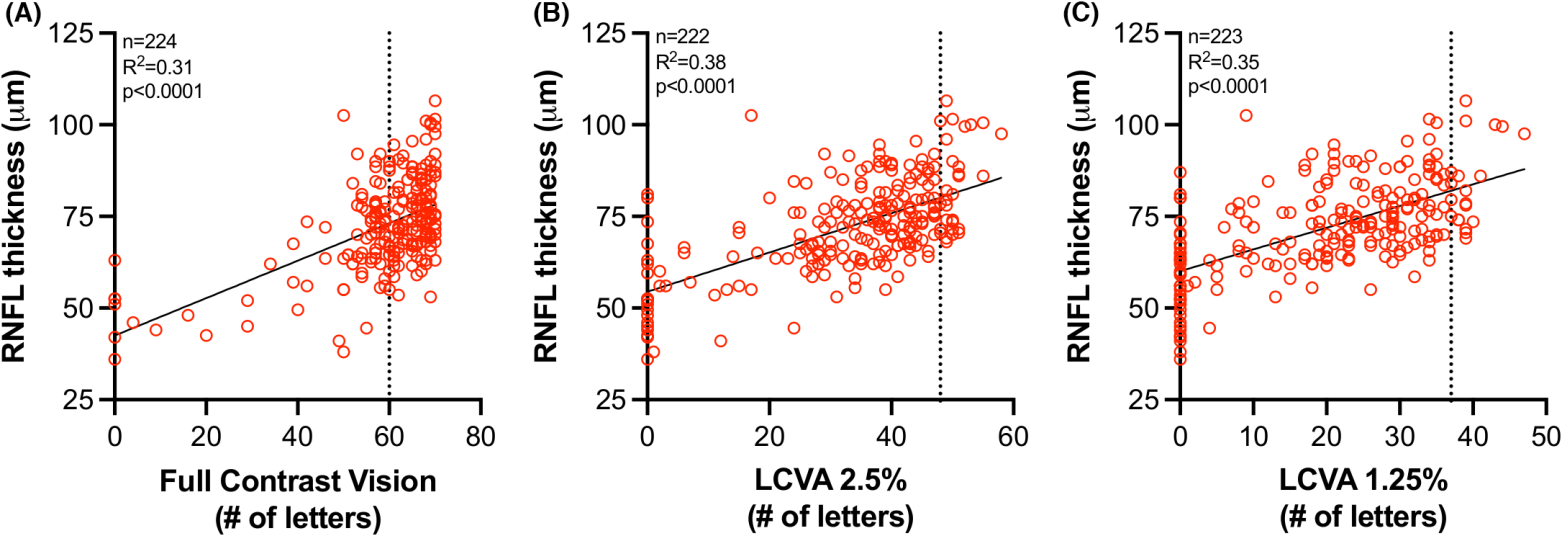
RNFL thickness predicts vision loss in FRDA. RNFL thickness plotted against (A) full‐contrast vision, (B) low‐contrast visual acuity (LCVA) at 2.5% contrast, and (C) LCVA at 1.25% contrast. Dotted line in (A) at *X* = 60 represents 20/20 vision. Dotted line in (B) and (C) represents values obtained from 4 non‐FRDA controls. The equation of linear regression curve in (A) is *Y* = 0.5107*X + 42.50, when *X* = 59 letters (vision below 20/20), *Y* = 73 μm. When *X* = 50 letters (2 lines lost compared to 20/20 vision), *Y* = 68 μm.

To determine the relationship between retinal pathology and functional vision, we compared RNFL thickness to visual acuity. RNFL thickness significantly predicted visual acuity in both high‐contrast and low‐contrast visual acuity testing (Fig. 6A–C). Reading 60 or more letters at high contrast is representative of 20/20 vision on the Sloan letter charts; consequently, reading fewer than 60 letters marks the beginning of clinical vision loss. While the majority of participants scored 60 or more letters at high contrast, the majority of patients scored below the non‐FRDA control scores in the low‐contrast vision tests (Fig. 6B,C). Using the equation of the linear regression of RNFL thickness vs. visual acuity, high‐contrast vision scores of 59 letters resulted when RNFL thickness reached 73 μm and scores of 50 letters (representing 2 lines on the letter chart lost compared to 20/20 vision) when RNFL thickness reached 68 μm (Fig. 6A). Using the equation of linear regression from Figure 4D (*Y* = −0.001405*X + 84.87), we calculated that RNFL values reached this threshold thickness of 68 μm at a disease burden of approximately 12,000 disease years, translating to a disease duration of approximately 17 years for participants with 700 GAA triplets (approximate median repeat length in this cohort) on GAA1 (Table 2). A person with age of onset at 11 years (median value in this cohort) could expect significant vision loss to begin in the third decade of life (age = 11 years + disease duration of 17 years = 28 years old).

**Table 2:** Disease duration when disease burden equals 12,000.

GAA1	Disease duration (years)
100	120
200	60
300	40
400	30
500	24
600	20
700	17
800	15
900	13
1000	12
1100	11
1200	10
1300	9
1400	9
1500	8

Disease burden = GAA1 multiplied by disease duration. The years of disease duration at a disease burden of 12,000 (the disease burden at which vision drops significantly below 20/20) are shown for GAA1 lengths of 100 to 1500 triplets.

### RNFL thickness in participants with point mutations

OCT also proved valuable in evaluation of participants with point mutations. As for subjects with two expanded GAA repeats, OCT and visual function decreased with increasing disease duration, and OCT values correlated with vision scores. Interestingly, although subjects with point mutations commonly develop visual loss,^25^, ^26^ not all point mutations developed substantial RNFL loss: Patients with G130V and I154F mutations generally fell above the line marking participants with 2 expanded GAA repeats indicating less severe retinal pathology (Fig. S4). In contrast, other specific mutations (L106S and 165 + 5G > C) almost uniformly had visual loss and decreased RNFL. This was particularly noticeable for participants with the latter mutation, who develop vision loss, RNFL loss and diabetes, but have generally spared arm function, speech, and cardiac function (Table S2).^27^ This suggests a specific phenotype associated with this mutation including diabetes and vision loss in association with ataxia, analogous to the specific phenotype of FRDA participants harboring the G130V.^25^, ^26^

### RNFL thickness decreases over time in FRDA

**Figure 7:**
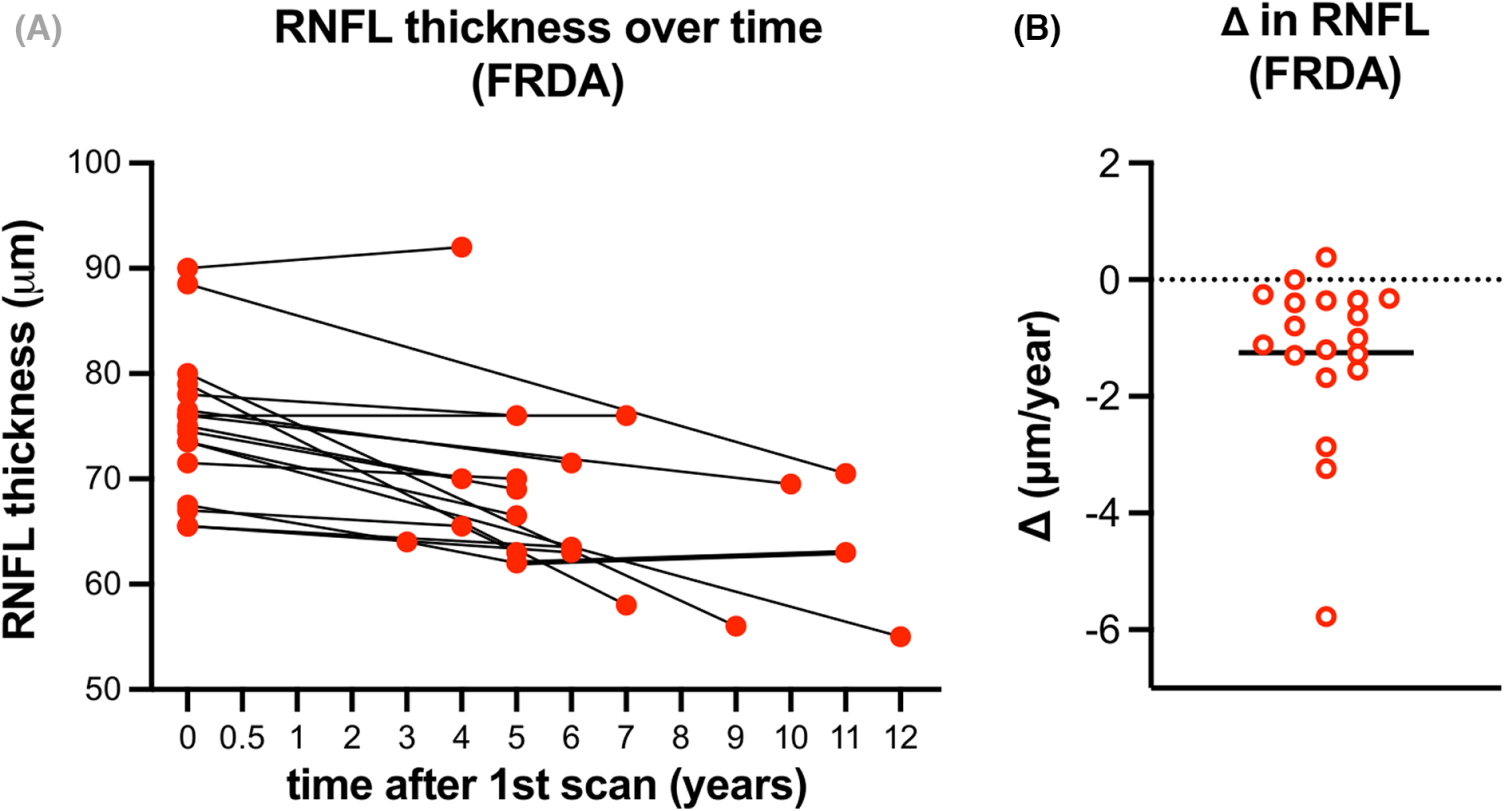
RNFL loss is −1.2 ± 1.4 um/year in FRDA. (A) RNFL thickness plotted over time in *n* = 17 people with FRDA. (B) Change in RNFL thickness (μm/year). Horizontal solid line depicts the mean (−1.2 ± 1.4 um/year).

RNFL thickness decreased with increasing disease burden, suggesting a decrease in RNFL thickness over time in a cross‐sectional analysis. To directly analyze change in RNFL thickness over time, we performed a longitudinal analysis. Sixteen participants with FRDA in our cohort had multiple OCT scans that were obtained at least 1 year apart. Most subjects showed a decrease in RNFL thickness over time (Fig. 7A). The median change in RNFL thickness was −1.2 ± 1.4 μm/year, and the range was −5.8 to 0.4 μm/year (Fig. 7B). In this small sub‐cohort, the rate of change did not correlate with GAA1 length, age, disease burden, or RNFL thickness at the time zero scan (not shown). Further, the rate of change in RNFL thickness was not different between those with >700 triplets on GAA1 and those with <700 triplets (not shown).

## Interpretation

We analyzed RNFL thickness in a cohort of 198 people with FRDA, including 61 children, and we found that RNFLs are pathologically thin in FRDA. Surprisingly, unlike most clinical measures of neurological progression in FRDA in which children present only minimally affected or even un‐affected, RNFLs were significantly thin around the time of presentation. Overall, many children with FRDA had thin RNFLs compared to age matched controls, which is suggestive of hypoplasia rather than simply degeneration.^28^ This finding resembles observations from somatosensory systems in FRDA, in which large fiber sensory function is largely absent at presentation and further promotes the increasingly supported hypothesis of a developmental contribution to FRDA pathophysiology.^29^, ^30^, ^31^

In addition, RNFL thickness was heterogenous among subjects and was predicted by disease burden such that subjects with longer GAAs and longer disease duration had thinner RNFLs, an indication of further degeneration over time. The longitudinal analysis showed that the rate of change was ~1 μm/year, double the rate of reported values for healthy controls.^32^ Thus, both hypoplasia and degeneration may contribute to the decreased RNFL thickness noted in FRDA. We did not, however, find any other predictors of the rate of RNFL thinning apart from sex, which has been reported as a modifier among healthy subjects in other studies.^33^ Our results showing that males have thinner RNFLs than females may be in contrast to findings from a previous study that showed that neurological symptoms of FA progress slightly faster in females compared to males. Taken together, the difference in RNFL thickness between males and females seems more likely due to normal differences in RNFL thickness between the sexes than it does with FA pathology. The presence of significantly decreased RNFL in some children but not others, suggest the possibility of modifier effects as well. More longitudinal data are needed to determine which patients will experience larger decreases in RNFL thickness over time.

RNFL thickness also predicted visual acuity. Using the equations of linear regressions, the threshold RNFL thickness at which subjects started exhibiting significant high‐contrast vision loss (50 letters or less on the Sloan letter charts) was 68 μm. RNFL thickness decreased over time and reached 68 μm at a disease burden of approximately 12,000 disease years, which equates to a disease duration of 17 years for a person with 700 GAA triplets on GAA1. Assuming a typical age of onset of approximately 11 years, a person with 700 GAA triplets on GAA1 could expect to start experiencing vision loss in the third decade of life. This manner to determine the age at which those with FRDA will start experiencing high‐contrast vision loss is only an approximation because it only considers two contributing factors, disease duration and GAA1 length, providing an *R* ^2^ of 0.32 (Fig. 4D), leaving room for other factors that were not uncovered in this study. Interestingly, RNFL thickness was a predictor of neurologic status as assessed by mFARS and FA Functional Disease Stage; the *R* ^2^ value for such correlations is higher than that noted for other CNS imaging measures, perhaps reflecting the global components of neurodegeneration in FRDA.^34^, ^35^, ^36^, ^37^, ^38^ While there are some differences in the mFARS and other neurological scales, that is the SARA^39^ the scales are collinear and overwhelmingly similar. Because mFARS is the standard neurological scale used in US clinical trials and the only neurological scale used in the FACOMS study that we sourced data from, we chose to use mFARS for this study. Future studies could focus on other scales, but given the similarities in such scales, we would not expect a different conclusion that the one from this study.

In this study, the majority of those with FRDA have pathologically thin retinas early in disease (even at presentation) despite having intact high‐contrast vision. The present findings suggest a need for more extensive longitudinal studies with adults and children with FRDA and reveal an unmet need for vision‐directed drug development including small molecules or gene therapy to treat vision loss in FRDA.^40^, ^41^

## Author Contributions

LNR, LJB, AND, and DRL contributed to the conception and design of the study. LNR, KM, MK, MW, CP, VP, AW, and CR contributed to the acquisition and analysis of data. LNR and DRL contributed to drafting the text and preparing the figures.

## Conflict of Interest

Nothing to report.

## Supporting information


Appendix S1.
