# Supplementary material for: Retinal hypoplasia and degeneration result in vision loss in Friedreich ataxia
Source: Ann Clin Transl Neurol. 2023 Jun 19;10(8):1397–406. doi: 10.1002/acn3.51830 (PMC10424660; doi:10.1002/acn3.51830)
Supplement: Supplementary file 1 — Appendix S1. [file ACN3-10-1397-s001.pdf]

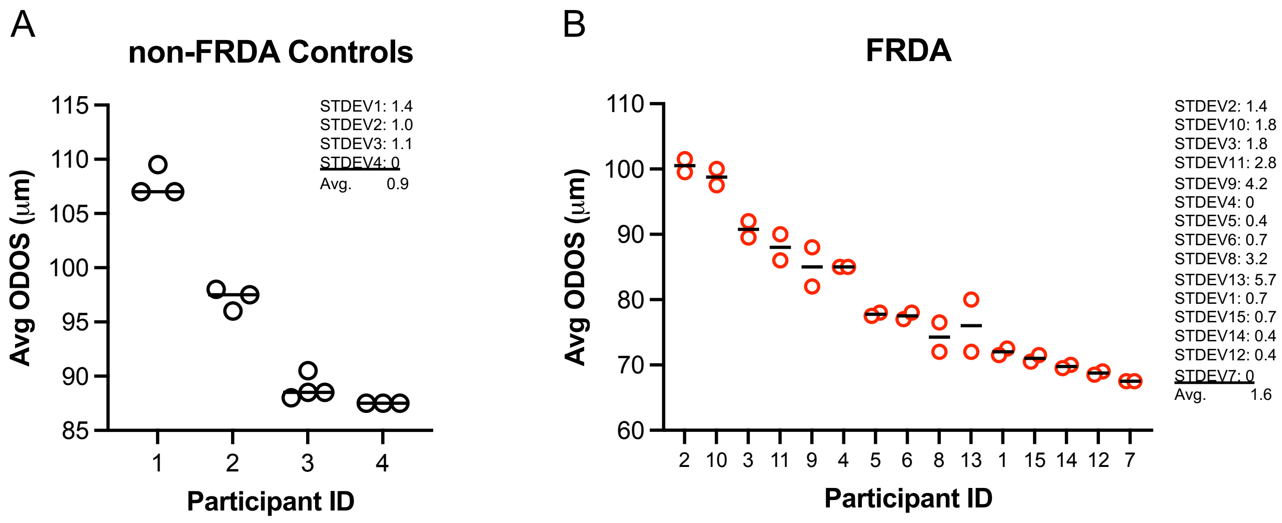

Figure S1. **Technical reproducibility in OCT measurements.** RNFL thickness for (A) 4 non-FRDA controls measured in triplicate and (B) 15 participants with FRDA in duplicate. Repeat scans were take <1 year apart. Standard deviations (STDEV) shown on right side of graphs. All scans were taken on a Cirrus OCT machine.

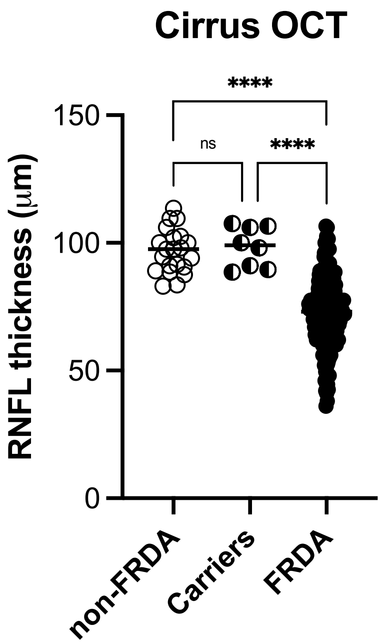

Figure S2. **Heterozygous carriers do not exhibit RNFL thinning**. Average RNFL thickness in both eyes from 21 non-FRDA controls, 8 heterozygous carriers of one GAA repeat expansion, and 198 participants with FRDA . Stats: one-way ANOVA with Tukey's post hoc analysis. n.s.=not significant, ****=p<0.0001.

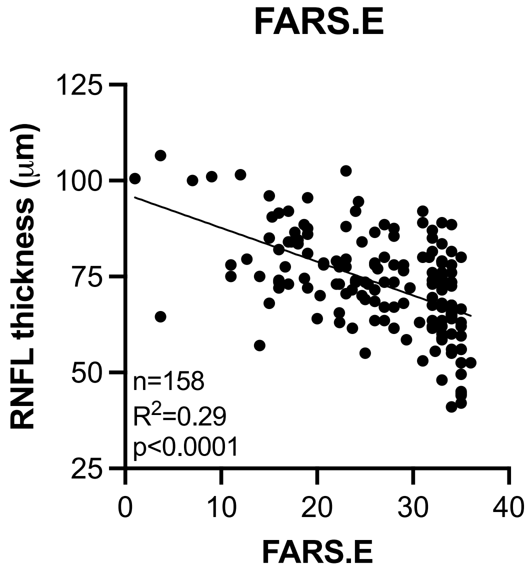

Figure S3. **RNFL thickness is predicted by the upright stability subscore of the mFARS**. Linear regression analysis shows a significant correlation between the upright stability subscore of the mFARS (FARS.E) and RNFL thickness in 158 participants with FA. The ceiling effect of this subscore can be seen at the upper end of the FARS.E on the X-axis.

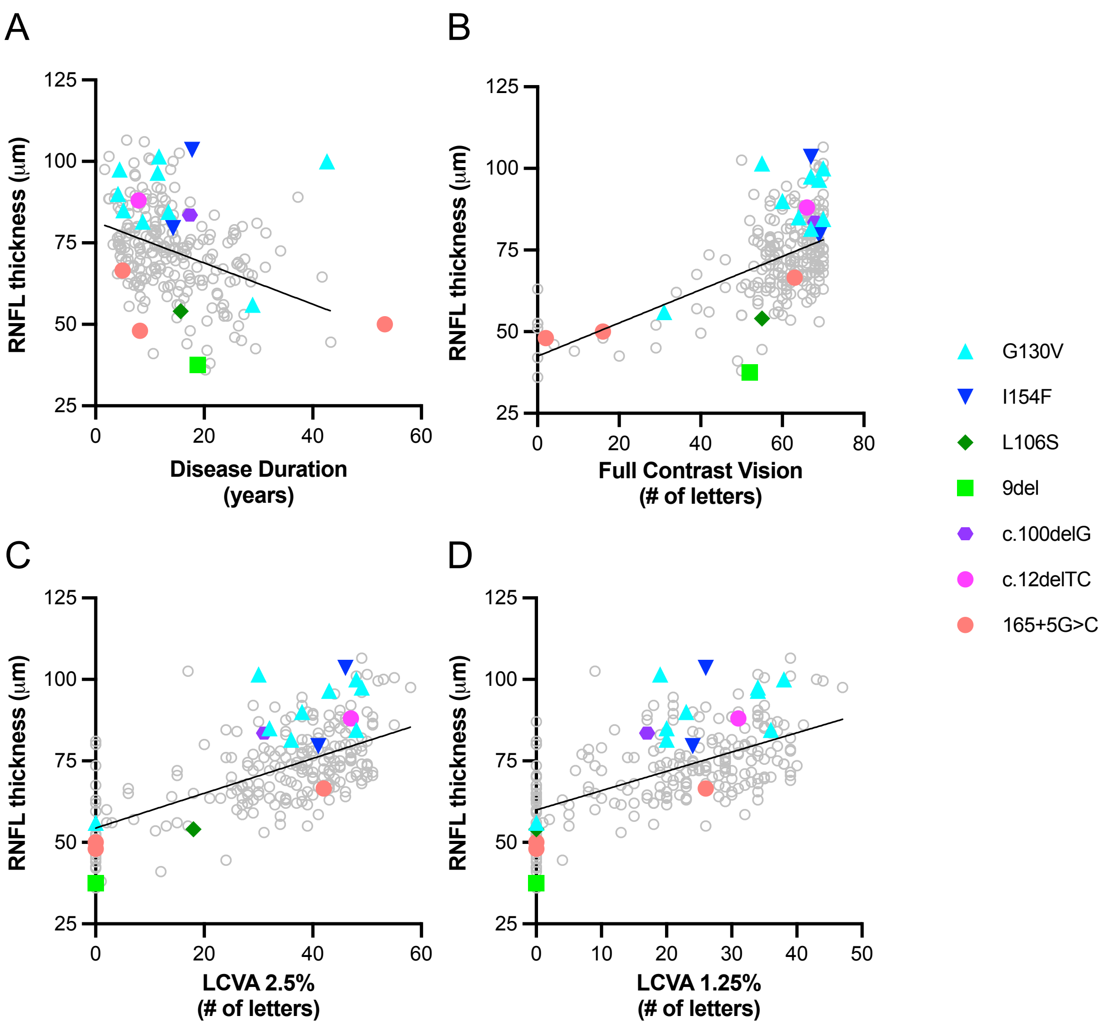

Figure S4. **RNFL thickness predicts vision loss in FRDA patients with point mutations, deletions, or other disease-causing variants of the *FXN* gene.** RNFL Thickness plotted against (A) disease duration, (B) full contrast visual acuity, (C) low contrast visual acuity (LCVA) 2.5%, and (D) LCVA 1.25% in people with FRDA that are homozygous for GAA expansion (GAA hom.) or various other disease-causing variants of the *FXN* gene (G130V, I154F, L106S, 9del, c.100delG, c.12delTC, and 165+5G>C.

	Quadrant	Avg RNFL Right (μm)	Avg RNFL Left (μm)	p-value
FRDA	Superior	84.92	86.28	0.46
	Inferior	93.75	92.34	0.49
	Nasal	55.83	57.40	0.22
	Temporal	56.84	59.22	0.06
CNTR	Superior	119.5	124.2	0.07
	Inferior	128.2	125.7	0.34
	Nasal	75.58	71.89	0.07
	Temporal	70.56	67.32	0.09

Table S1. Average RNFL thickness in each quadrant (superior, inferior, nasal, and temporal) for the right eye (OD) and the left eye (OS) for participants with FA. P-values from t-test comparing the averages for each quadrant.

	Age	GAA	AOO	Duration	RNFL	FCVA	LCVA 2.5%	AOO of DM	CM	mFARS	Speech	UE
1	11	1187	6	5	66.5	63	42	11	No	47/95	0/3	8/36
2	21	900	8	13	48	2	0	17	No	43/95	0/3	1/36
3	61	670	8	53	50	16	0	40	No	87/95	0/3	29/36

Table S2. Comparison of most recent evaluations for 3 participants with FRDA with one expanded allele and a 165+5G>C mutation on the second allele. This mutation presumptively disrupts splicing of the first intron. All are male. Subject 3 later died at age 63. All subjects showed loss of RNFL thickness, progressive loss of vision, diabetes, but sparing of speech, heart, and to a lesser extent arm function. The severity correlates with disease duration and GAA1 length.

AOO=age of onset, CM =cardiomyopathy, UE = Upper extremity mFARS score, FCVA=full-contrast visual acuity, LCVA 2.5%=low-contrast visual acuity
